# Network Pharmacology-Based Study of the Underlying Mechanisms of Huangqi Sijunzi Decoction for Alzheimer's Disease

**DOI:** 10.1155/2021/6480381

**Published:** 2021-10-05

**Authors:** Wei Zhang, Mingti Lv, Yating Shi, Yonghui Mu, Zhaoyang Yao, Zhijun Yang

**Affiliations:** ^1^School of Basic Medical Sciences, Xinxiang Medical University, Xinxiang 453003, China; ^2^School of Life Science and Technology, Xinxiang Medical University, Xinxiang 453003, China

## Abstract

**Background:**

Huangqi Sijunzi decoction (HQSJZD) is a commonly used conventional Chinese herbal medicine prescription for invigorating Qi, tonifying Yang, and removing dampness. Modern pharmacology and clinical applications of HQSJZD have shown that it has a certain curative effect on Alzheimer's disease (AD).

**Methods:**

The active components and targets of HQSJZD were searched in the Traditional Chinese Medicine Systems Pharmacology Database and Analysis Platform (TCMSP). The genes corresponding to the targets were retrieved using UniProt and GeneCard database. The herb-compound-target network and protein-protein interaction (PPI) network were constructed by Cytoscape. The core targets of HQSJZD were analysed by Gene Ontology (GO) and Kyoto Encyclopedia of Genes and Genomes (KEGG). The main active compounds of HQSJZD were docked with acetylcholinesterase (AChE). In vitro experiments were conducted to detect the inhibitory and neuroprotective effects of AChE.

**Results:**

Compound-target network mainly contained 132 compounds and 255 corresponding targets. The main compounds contained quercetin, kaempferol, formononetin, isorhamnetin, hederagenin, and calycosin. Key targets contained AChE, PTGS2, PPARG, IL-1B, GSK3B, etc. There were 1708 GO items in GO enrichment analysis and 310 signalling pathways in KEGG, mainly including the cAMP signalling pathway, the vascular endothelial growth factor (VEGF) signalling pathway, serotonergic synapses, the calcium signalling pathway, type II diabetes mellitus, arginine and proline metabolism, and the longevity regulating pathway. Molecular docking showed that hederagenin and formononetin were the top 2 compounds of HQSJZD, which had a high affinity with AChE. And formononetin has a good neuroprotective effect, which can improve the oxidative damage of nerve cells.

**Conclusion:**

HQSJZD was found to have the potential to treat AD by targeting multiple AD-related targets. Formononetin and hederagenin in HQSJZD may regulate multiple signalling pathways through AChE, which might play a therapeutic role in AD.

## 1. Introduction

Alzheimer's disease (AD) is a neurodegenerative disease accompanied by progressive memory and cognitive decline [1, 2]. Amyloid-beta (A*β*) protein and the microtubule-associated protein Tau are recognised as two important elements involved in the development of AD [3]. At present, the most commonly used drugs in the clinical treatment of AD are cholinesterase inhibitors, such as Donepezil, Rivastigmine, Galantamine, and Aricept, which only improve some symptoms of AD by increasing acetylcholine (Ach) [4]. It is essential to identify new drugs and treatments to prevent AD.

An increasing number of studies have proven that traditional Chinese medicine (TCM) plays a very important role in the treatment of complex diseases [5, 6]. Huangqi Sijunzi decoction (HQSJZD) is commonly used in TCM and includes five kinds of traditional Chinese herbs: *Hedysarum Multijugum* Maxim (“Huangqi” in Chinese, HQ), *Panax Ginseng* C. A. Mey (“Renshen” in Chinese, RS), *Atractylodes Macrocephala* Koidz (“Baizhu” in Chinese, BZ), *Poria Cocos* S. Wolf (“Fuling” in Chinese, FL), and *Licorice* (“Gancao” in Chinese, GC). As the major component of HQSJZD, HQ plays a very effective role in the treatment of many diseases. HQ has an optimal therapeutic effect on cholinergic substances, the brain, immune regulation, and cancer when combined with different TCMs [7–9]. RS also plays an important role in HQSJZD and is beneficial to Qi, preventing ageing and prolonging life. BZ, as a medicinal plant, has long been used as a tonic in various ethnic medical systems to treat gastrointestinal dysfunction, cancer, osteoporosis, obesity, and foetal irritability [10, 11]. FL has diuretic, sedative, and tonic effects in traditional Chinese and Japanese medicine. FL is widely used as a component of many preparations in Asian medicine because of its significant anti-inflammatory activity and ability to inhibit angiogenesis in acute and chronic inflammation [12, 13]. According to the classical theory of TCM, “nine out of ten prescriptions contain *licorice*.” GC being one of the most important Chinese herbal medicines in TCM and can reduce toxicity and improve the efficacy of some Chinese herbal medicines in some prescriptions [14, 15]. These five kinds of traditional Chinese herbs complement each other in clinical treatments and are used to treat many diseases. However, TCM formulas are characterised by multiple components, multiple targets, and multiple pathways [6]. The therapeutic effect of HQSJZD on AD has not been fully elucidated, and it is necessary to determine its effect on AD to perform further systematic investigations.

Network pharmacology is a cutting-edge methodology for discovering new therapeutic drugs and understanding the therapeutic mechanisms of complex diseases [16]. In recent years, remarkable results have been achieved in exploring the mechanisms of action of TCM, screening the effective components of TCM, and searching for therapeutic targets of TCM [17–19]. Network pharmacology can be used as a bridge between Western medicine and traditional medicine and skilfully connect them, which can greatly advance research on the mechanism of the synergistic effect of TCM.

Therefore, TCM network pharmacology establishes a link between the molecular targets of networks and disease patterns. We used network pharmacology to identify the effective natural components and key targets of HQSJZD, and then, molecular docking analysis was performed to verify the predicted molecular interactions between the compounds and their targets. Our study determined the relationship among the TCM, ingredients, and targets of HQSJZD and drugs, which is of great significance for discovering the potential mechanism of HQSJZD in the treatment of AD and for future pharmacological research on and clinical applications of HQSJZD. This study provides a new reference basis for HQSJZD.

## 2. Material and Methods

### 2.1. Collection of the Chemical Ingredients of HQSJZD

Using the Chinese pinyin “Huangqi, Renshen, Baizhu, Fuling, and Gancao” as keywords, data were collected from the Traditional Chinese Medicine System Pharmacology Database (TCMSP, https://tcmspw.com/index.php), which is a systematic pharmacology database and analysis platform for TCM. According to the requirement of their high utilisation in TCM, eligible compounds were selected from the above active components [20]. The disease information in TCMSP comes from the Therapeutic Target Database (TTD, https://db.idrblab.org/ttd/), which can be queried and downloaded. Moreover, TTD also provides pharmacokinetic information for each compound, and compounds with good drug-like and ADME (absorption, distribution, metabolism, excretion) characteristics can be selected for further research. To identify drugs with good absorption and chemical suitability, two ADME-related parameters, oral bioavailability (OB) ≥30% and drug-likeness (DL) ≥0.18, were employed to screen potential bioactive ingredients using TCMSP in this study [21].

### 2.2. Collection of Compound-Related Targets and AD-Related Targets

With “Alzheimer's disease” as the keyword, GeneCard (https://www.genecards.org/), DrugBank (http://www.drugbank.ca), and TTD were searched and screened for AD-related targets. Then, the corresponding gene names were found in the UniProt knowledge base (UniProtKB) (https://www.uniprot.org/) by searching for “*Homo sapiens*” [22].

### 2.3. Network Construction and Central Network Topological Analysis

We used Cytoscape 3.6.1 software to generate a network diagram of the interactions between active components and targets and the interactions between targets and targets [23]. Topological analysis was performed by the network analyzer module of Cytoscape 3.6.1, which is a useful tool for analyzing and visualizing biological networks and is currently the most reliable software.

The topological properties of the protein-protein interaction (PPI) network were analyzed by using Cytoscape 3.6.1. “Nodes” were used to represent target protein molecules, “edges” were used to represent mutual relationships, and the intermediary center of the PPI network was calculated according to betweenness centrality, closeness centrality, and degree, so that the interaction between each component and the target could be displayed [24].

### 2.4. Target Prediction

Using Venn software, the screened targets corresponding to the active ingredients of HQSJZD were crossed with the disease targets corresponding to AD targets, and common targets were obtained through a Venn diagram. Then, the prediction targets were imported into the STRING database (https://string-db.org/) for further predictions.

### 2.5. GO and KEGG Pathway Enrichment Analysis

The Metascape database (https://metascape.org/) is an online biological knowledge base and an analytic tool used to extract biological information regarding gene functional classification, functional annotation, and enriched pathways [25]. The gene names of the predicted potential targets were imported into the Metascape database for GO and KEGG pathway analyses [26–28]. GO terms (BP: biological process, CC: cell component, and MF: molecular function) with *P* < 0.05 and KEGG pathways with *P* < 0.05 were considered being significant [29].

### 2.6. Molecular Docking

Molecular docking was performed to determine the binding affinity of the compounds of HQSJZD with AChE. Through a tool used to edit binding sites and search for binding sites that involve receptors and ligands based on information from the RCSB protein database (PDB) (http://www.pdb) or the sites of the original ligands of proteins, crystal structures were obtained [30]. Then, 3D structures of acetylcholinesterase (AChE) (PDB Code: 4ey7) were got from the PDB database in PDB format by setting the organism to “*Homo sapiens*.” Three-dimensional conformers of the candidate compounds were gained from the PubChem database (https://pubchem.ncbi.nlm.nih.gov/) in MOL format [31]. Subsequently, they were imported into AutoDock Tools 4.4.6 after removing water and small molecules by the molecular structure tool. The protein was set as a rigid filename; the compound was set as flexible; the docking mode adopted the genetic algorithm; docking was performed by Lamarckrian (4.2); the binding pocket parameters were set based on access to information and literature, as the grid *X*, *Y*, and *Z* coordinates were −6.141, −43.015, and 26.199 [32]; the other parameters were set to the default values. The highest negative binding energy was considered the ligand molecule with maximum binding affinity. All the obtained conformations of AChE and the compounds were analyzed to determine the interactions and binding energy of the docked structure using PyMOL molecular visualisation software.

### 2.7. In Vitro AChE Activity Inhibitory Assay

AChE activity was assessed by the improved Ellman's method [33]. Donepezil, an AChE inhibitor widely used to treatment of AD, was used as the reference compound. The AChE assay kit was bought from Nanjing Jiancheng Bioengineering Institute, and AChE was obtained from brains of the SD rats. The blood vessels were removed from brains, and then, the brains were homogenized with 10-fold 0.9% saline solution on the ice. After being centrifuged for 10 min at 2500 ×g at 4°C, the supernatant from brain homogenate was collected. To determine the inhibition of AChE activity, six serial dilutions of samples were added. According to the kit operation, the 250 *µ*l acetylcholine substrate, 250 *µ*l developer, 50 *µ*l test solution of different concentrations, and 50 *µ*l of enzyme solution were added to the test tube. And after being incubated for 6 min at 37°C, add 15 *µ*l until the inhibitor terminates the reaction. Then, the absorbance intensity of AChE reaction system was quantified at 412 nm by enzyme-labeled instrument (SoftMax@ Pro7 Software, Shanghai, China). Experiments were in accordance with guidelines for animal care and were approved by the Institutional Animal Care and Utilization Committee of Xinxiang Medical University.

### 2.8. Cell Cultures and Cell Viability Assay

Human neuroblastoma SH-SY5Y cells were maintained in a medium consisting of high glucose DMEM supplemented with 10% fetal bovine serum (FBS) in humidified 5% CO_2_ at 37°C. SH-SY5Y cells were plated at a density of 4,000 cells per well in 96-well plates and cultured for 16 h. To determine the neuroprotective effect of active constituents on SH-SY5Y cells, cells were pretreated with 0.5, 1.0, 1.5, and 2 *μ*M formononetin in HQSJZD for 3 h and then treated with sodium nitroprusside (500 mM) or potassium chloride (80 mM) for 20 h, respectively.

Cell viability was assessed by cell counting kit-8 (CCK-8) assay according to the manufacturer's instructions. In short, after treatment of sodium nitroprusside (Aladdin, China), or potassium chloride (Aladdin, China), 10 *μ*L of CCK-8 reagent (Biosharp, Shanghai, China) was subsequently added to each well for 3 h incubation at 37°C. Finally, cell viability was determined by reading the optical density (OD) at a wavelength of 450 nm using an enzyme-labelled instrument (SoftMax@ Pro7 Software, Shanghai, China).

### 2.9. Statistical Analysis

All data are presented as mean ± SEM. Statistical analysis was carried out using the GraphPad Prism version 7.0 software, and the significance of each group was verified with one-way analysis of variance (ANOVA) followed by Tukey's multiple comparison post hoc test. A *P* value <0.05 was considered significant.

## 3. Results

### 3.1. Screening of the Targets and Active Components of HQSJZD

**Figure 1:**
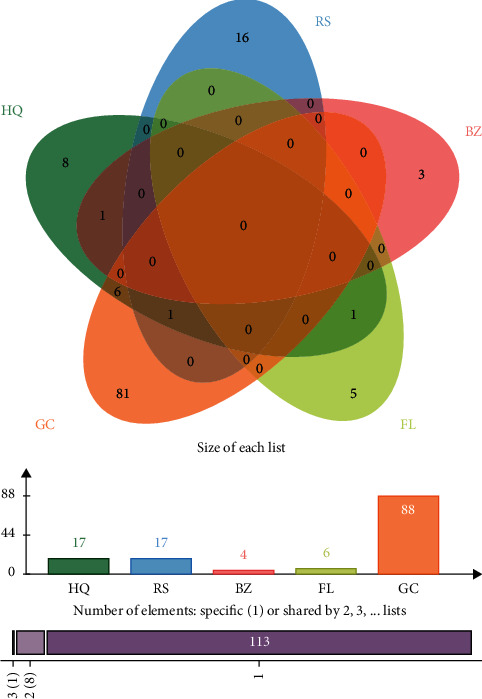
Composition distribution of HQSJZD decoction.

A total of 132 active components were isolated and identified in HQSJZD using the TCMSP database (Figure 1). According to the literature, 17 active components were identified from HQ and RS, 88 from GC, 4 from BZ, and 6 from FL. HQ, as the main component of HQSJZD, was taken as an example, and the 17 active components of HQ are presented in Table 1.

A total of 255 compound-related targets were identified from the databases after eliminating duplicates. There were 210 targets in HQ, 115 in RS, 18 in BZ, 24 in FL, and 230 in GC, and the composition of jaranol in HQ was used as an example and is presented in Table 2.

### 3.2. Construction of the Active Ingredient Gene Target Network

**Figure 2:**
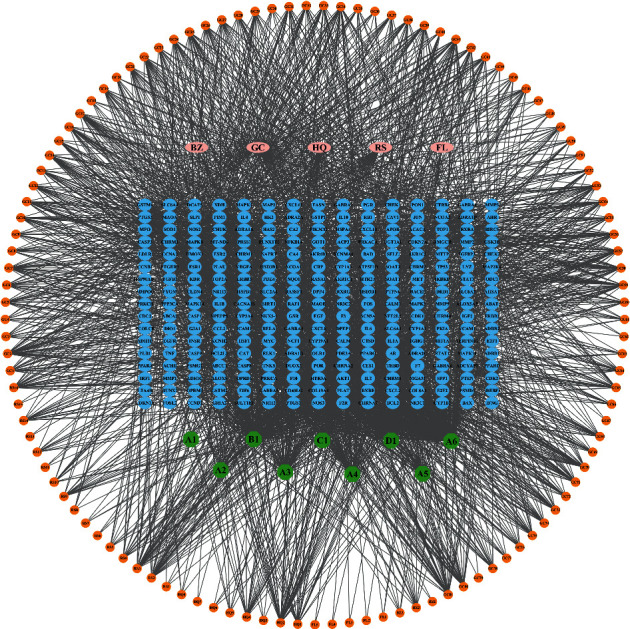
Herb-compound-target network of HQSJZD. Blue nodes: protein targets; circular nodes: compounds; edges: interactions between compounds and proteins.

To clarify the relationship among the TCM, active ingredients, and potential targets of HQSJZD, the target network of HQSJZD was constructed, as shown in Figure 2. This network comprises 382 nodes and 2655 edges, and the pink target in the middle is the active component of TCM in HQSJZD, and the blue target is the potential prediction target of TCM, and A1–6 (mairin, jaranol, isorhamnetin, formononetin, calycosin, and quercetin) are the common components of HQ, GC, and B1 (kaempferol), which are the common ingredients of HQ, GC, and RS. C1 ([(3S,8S,10R,13R,14S,17R)-10,13-dimethyl-17-[(2R)-6-methylheptan-2-yl]-2,3,4,7,8,9,11,12,14,15,16,17-dodecahydro-1H-cyclopenta[a]phenanthren-3-yl] 2,4-dichlorobenzoate) is a common component of HQ and BZ, and D1 (hederagenin) is a common component of HQ and FL.

### 3.3. Construction of the Key Ingredient AD Target Network

**Figure 3:**
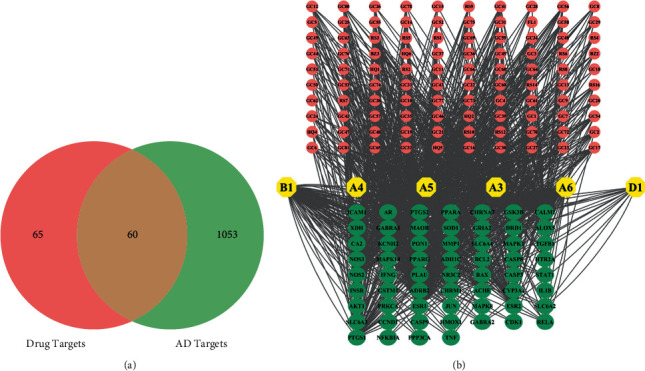
(a) Intersection of drug targets and AD disease targets; the number represents the number of targets. (b) Compound-target network of HQSJZD. Green nodes: protein targets; yellow nodes: key compounds; Orange pink: the ingredients of Chinese medicine; Edges: interactions between compounds and proteins.

The preliminary screening of 125 targets and AD-related targets intersected, and 60 predicted key targets were got by Venn software in (Figure 3(a)). We constructed the key components and AD target network of HQSJZD in (Figure 3(b)). The upper part of the square arrangement shows the corresponding targets of active components, and the lower part of the square arrangement shows the 60 predicted targets in HQSJZD. The quantitative property “degree” was calculated as the number of edges linked to each node, showing the importance of a node in a network. Radiality is a node centrality index computed by subtracting the average shortest path length of a node *n* from the diameter of the connected component plus 1. The radiality of each node is divided by the diameter of the connected component. Thus, it is a number between 0 and 1. Topological Coefficient attribute stores the topological coefficient of *n*. Nodes with less than 2 neighbors have a topological coefficient of zero [34]. Topological analysis shows that quercetin, kaempferol, formononetin, isorhamnetin, hederagenin, and calycosin play important roles in HQSJZD. The specific results are shown in Table 3.

### 3.4. GO and KEGG Pathway Enrichment Analysis

**Figure 4:**
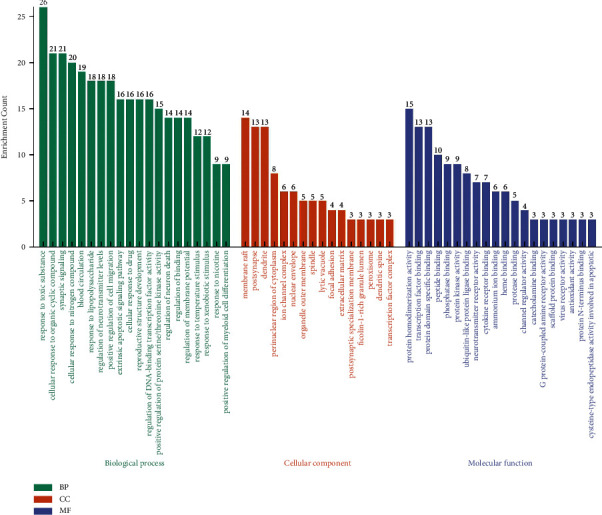
GO enrichment analysis of the 60 targets of HQSJZD. The chart is arranged in order from high to low according to the number of target distributions.

GO and KEGG pathway enrichment analysis were performed to elucidate the characteristics of the HQSJZD-related targets. The number of GO items was 1708 (*P* < 0.05), including 1510 items for BP, 73 items for CC, and 125 items for MF. GO analysis showed that most of the potential targets were found in CC, including membrane rafts, postsynaptic complexes, cytoplasmic complexes, the organelle outer membrane, and ion channel complexes, and had good binding properties with neural receptors, enzymes, and peptides. Specifically, in BP, most of the target proteins had rich functions in toxic reactions, drug reactions, synaptic signals, neurotransmitter transmission, neuronal death regulation, blood circulation, proliferation and differentiation regulation, and exogenous apoptosis signalling processes. MF was found to interact with transcription factors, as well as a specific protein, and showed good antioxidant activity. These results indicate HQSJZD has multiple synergistic effects in biological processes (Figure 4).

**Figure 5:**
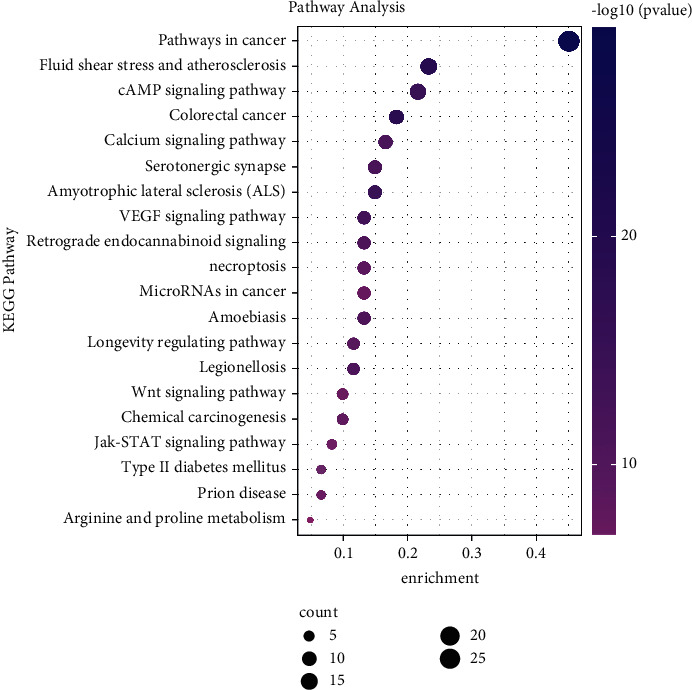
KEGG enrichment analysis of the potential targets of HQSJZD. The larger rich factor stands for the higher level of enrichment. The size of the dot denotes the number of target genes in the pathway, and the color shade of the dot indicates the different *P* value range.

Furthermore, KEGG enrichment analysis showed that 310 pathways (*P* < 0.05) were affected by HQSJZD. The top 10 enriched pathways included pathways involved in cancer, colorectal cancer, amyotrophic lateral sclerosis (ALS), cAMP signalling, vascular endothelial growth factor (VEGF) signalling, serotonergic synapses, calcium signalling, legionellosis, amoebiasis, and retrograde endocannabinoid signalling (Figure 5).

### 3.5. Potential HQSJZD Target-AD Target Network

**Figure 6:**
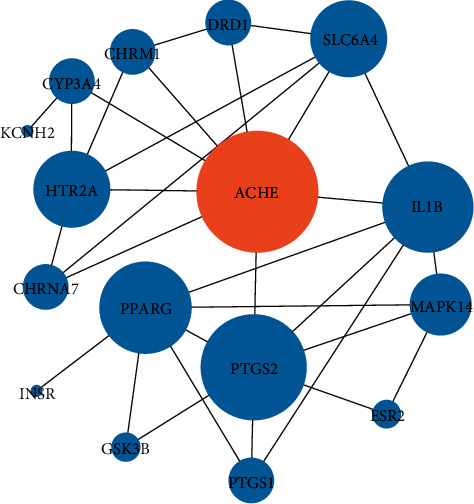
Protein-protein interaction network (network of the 16 key targets based on the STRING database. Large size represents higher degree value).

We compared the final 60 predicted targets with clinically applicable targets of AD and found 20 common targets. The key targets included AChE, PTGS2, PPARG, and GSK3B. We imported these 20 targets into the STRING database and selected “*Homo species*” to explore their interactions. Sixteen of the targets had strong interactions (confidence score ≥5) in the PPI map generated from the STRING database (Figure 6). We found AChE represented the key index for HQSJZD in the treatment of AD based on degree and middle centrality.

### 3.6. Binding Capacity of the Active Compounds of HQSJZD and AChE

**Figure 7:**
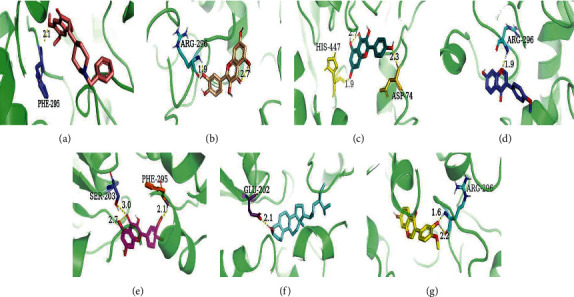
Results of docking 6 compounds with AChE. (a) Donepezil; (b) quercetin; (c) kaempferol; (d) formononetin; (e) isorhamnetin; (f) hederagenin; (g) calycosin. The yellow dotted line: hydrogen bond; the number: bond distance.

**Figure 8:**
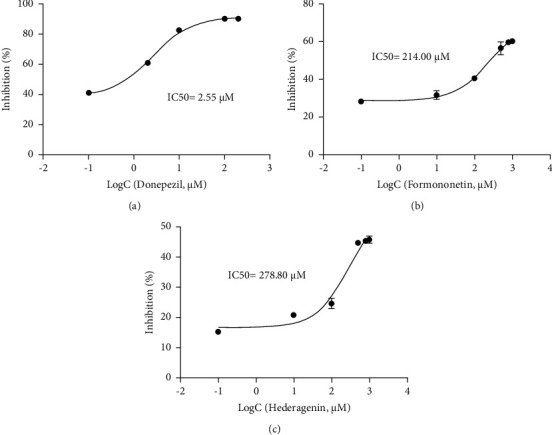
The inhibitory effect of donepezil (a), formononetin (b), and hederagenin (c) on AChE activity. Results are presented as means ± SEM, *n* = 3.

AChE is one of the prime targets of HQSJZD in the treatment of AD, and it is also one of the drug targets in the treatment of AD [35, 36]. The predictive compounds, including kaempferol, formononetin, hederagenin, quercetin, isorhamnetin, and calycosin, were the top 6 compounds (ranked by degree score) in the compound-target network of HQSJZD. Donepezil docked with AChE as a positive control. Molecular docking was used to verify if these six compounds had a significant role in regulation of AChE. The result showed that all the key compounds in the network had a strong affinity with AChE protein. Hederagenin and formononetin are bound to the active pocket of the target via hydrogen bonds and hydrophobic interactions, which may lead to better activity (Figure 7). In vitro experiments further evaluate the effects of these six compounds with AChE. The formononetin and hederagenin were found to display general AChE inhibitory activity (60.15% and 45.72%, respectively), compared to that of the reference compound donepezil (90.18%). Quercetin and kaempferol do not inhibit AChE, while isorhamnetin had weak inhibitory activity on AChE (Table 4). Donepezil was with an IC50 value of 2.55 *μ*M. Among the six constituents, two constituents of formononetin and hederagenin were identified to exhibit moderate inhibitory activity toward AChE, with the IC50 values of 214.00 *μ*M and 278.80 *μ*M, respectively (Figure 8). HQSJZD has more ingredients and complex mechanism. Formononetin has a higher inhibition rate and deserved the further study. Therefore, we tested the neuroprotective effects of formononetin.

### 3.7. Neuroprotective Effect of Active Constituents in HQSJZD

**Figure 9:**
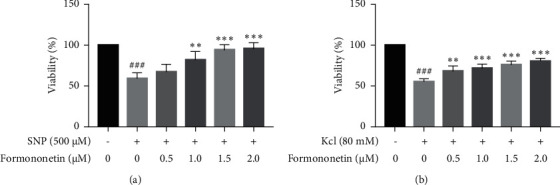
The protective effects of formononetin against cell injury induced by sodium nitroprusside (a) and potassium chloride (b) in SH-SY5Y cells. Results are presented as means ± SEM, *n* = 3. ^###^*P* < 0.001 versus each control group. ^*∗*^*P* < 0.05, ^*∗∗*^*P* < 0.01, ^*∗∗∗*^*P* < 0.001, versus group solely treated with sodium nitroprusside or potassium chloride, respectively.

As formononetin was the most active compound with AChE inhibitory activity among the available 6 compounds got from HQSJZD database, it was then chosen for the further in vitro cell experiments to assess its actual neuroprotective effects. Compared with the control group, the viability of cells preincubated with compounds was reduced while under the sodium nitroprusside or potassium chloride treatment. Cell viability was significantly improved in the groups pretreated with 0.5 *μ*M, 1 *μ*M, 1.5 *μ*M, and 2 *μ*M formononetin than in the group treated with sodium nitroprusside and potassium chloride alone (Figure 9). Moreover, the neuroprotective effect of formononetin on SH-SY5Y cells against sodium nitroprusside induced toxicity was more obvious than that in the potassium chloride induced cell injured models.

## 4. Discussion

Network pharmacology is a promising method for drug discovery and development, especially for TCM research [37]. Network pharmacology provides a new “multicomponent, multitarget, multieffect” network model that agrees with the characteristics of TCM and the holistic view of TCM treatment of AD [38]. From the perspective of TCM, the aetiology of AD is caused by a deficiency of the kidney essence and memory loss, and after the emergence of various diseases, toxic pathogens, such as blood stasis, block the meridians and collaterals, leading to dementia [39]. HQSJZD is a compound used to tonify Qi and blood, invigorate the spleen, and calm the mind, and HQSJZD may treat AD. Our study aimed to evaluate the mechanisms of the constituents of HQSJZD for the potential treatment of AD by using the network pharmacology method.

A total of 255 targets and 16 predicted key targets were obtained by assessing the target proteins of HQSJZD in various bioinformatics databases and the generated composite target network. APP and AChE are principally neuronal membrane-bound proteins. APP is the precursor of A*β* peptide (pathological factor of AD), and AChE can increase cholinergic defects or aggravate A*β* fibril formation and toxicity [40]. We found these targets had a strong correlation with AD and played important roles. For instance, AChE is a key target enzyme in AD, as shown in many clinical studies of mental diseases, and can be designed to inhibit the reaction of some proteins or drugs [41, 42]. PTGS2, a microglial marker that is widely expressed in the human body, plays a key role in the neuroinflammatory response and has a certain correlation with AD [43, 44]. PPARG is a transcription factor that regulates genes related to fatty acid metabolism and controls the peroxisome *β* oxidation pathway of fatty acids, which can improve the clearance of toxic molecules, such as A*β*, by the brain [45]. GSK3-*β* plays a key role in the pathophysiology of AD and can significantly decrease the ability of MAPT/Tau to bind and stabilise microtubules [46]. The cholinergic hypothesis suggests AD arises due to the dysfunction of acetylcholine containing neurons in the brain, and most of the clinically used anti-AD drugs preserve acetylcholine inhibiting AChE. Thus, it will likely remain pivotal for rational drug development for the treatment of AD to target acetylcholine deficiency.

Most clinical cholinesterase inhibitors have achieved some promising results in the treatment of AD patients [47]. In our study, molecular docking was performed to verify the strong affinity between HQSJZD and AChE, and to systematically explore the mechanism of HQSJZD in the treatment of AD. This study predicted that 6 compounds, quercetin, kaempferol, formononetin, isorhamnetin, hederagenin, and calycosin, had certain therapeutic effects on AD. Quercetin, as shown in a variety of studies, has a positive effect on memory improvement and anti-AD activity and can treat mental illness. Kaempferol, a natural acetylcholinesterase inhibitor, can delay the loss of cognitive ability, improve memory impairment, and reduce oxidative stress and neuroinflammation. Formononetin plays a protective role against the HT22 cell neurotoxicity induced by A*β*25-35 by participating in the PI3K/Akt signaling pathway, increasing the activity of *α*-secretase and the release of sAPP*α*, accelerating the non-amyloidosis process of amyloid precursor protein (APP), and reducing the production of A*β* [48]. Isorhamnetin, which is composed of quercetin and kaempferol derivatives, can protect the nervous system [49]. In addition, isorhamnetin (IRA) has a protective effect on A*β*-induced cytotoxicity in human neuroblastoma SH-SY5Y cells. The results of an in vitro A*β* aggregation assay suggested that IRA destabilizes A*β* fibrils [49]. Flavonoids are very useful in identifying potential drugs for treating various neurodegenerative diseases, including AD [50]. Hederagenin, as a component of *Salvia miltiorrhiza*, has been proven to inhibit AChE and butyrate cholinesterase (BChE) in vitro [51]. It has been reported that calycosin can alleviate oxidative stress and inflammation in the hippocampus of AD model mice by activating the protein kinase C pathway, thus improving cognitive function [52]. Therefore, the above predicted active ingredients indicate that HQSJZD may be effective in treating AD.

In order to clarify the relevant signal channel of HQSJZD on AD, we first performed GO enrichment analysis, only kept the first 20 significant enrichments, and found that HQSJZD treatment of AD involves multiple signaling pathway processes, including antioxidative stress process, neurotransmitter signal transmission process, cell proliferation and differentiation, and other processes that synergistically act. In the KEGG pathway database, someone significantly enriched them in several pathways associated with AD such as calcium signalling pathway, Wnt signalling pathway, and type II diabetes mellitus. The dysregulation in cellular calcium signalling pathway homeostasis is the principal driving force of neurodegeneration in AD [53]. Wnt signalling pathway can effectively regulate hippocampal synapses to improve learning and memory abilities, controlling the pathogenesis of AD [54]. Type II diabetes and AD are closely related, and cognitive impairment is their common comorbidity. It involved the oxidative stress pathway in this, which promotes the development of the two diseases [55, 56].

Molecular docking can reveal interactions between components and their targets in a network by their evaluating binding energy, improving the accuracy of the network [57]. Figure 7 shows the docking result of the 6 compounds and AChE, indicating that there was a good affinity between them [58]. Quercetin, isorhamnetin, kaempferol, formononetin, hederagenin, and calycosin are connected to the proteins in AChE by hydrogen bonds and hydrophobic interactions. These results strongly suggest that the active compounds in HQSJZD can effectively treat AD by binding AChE. Therefore, the available 6 compounds interacting with AD targets in HQSJZD were furtherly subjected to the in vitro AChE inhibitory assay to assess their inhibitory activity of AChE. Our results found that formononetin and hederagenin have moderate inhibitory effect on AChE. The mechanism of HQSJZD in treating AD is complicated. Then, we studied the effects of the major components on nerve cells.

To evaluate the neuroprotective effects of formononetin during the pathology progression of AD, two cell damaged models were established. As the NO donor, Sodium nitroprusside (SNP) causes neural damage. NO regulates the release of proinflammatory molecules, interacts with ROS leading to the formation of reactive nitrogen species (RNS), and targets vital organelles such as mitochondria, ultimately causing cellular death, a hallmark of AD [59]. Potassium chloride causes depolarisation of neuronal cells and promotes voltage-dependent calcium channels opening, leading to increases in intracellular calcium ion levels. Overloading of calcium ions in neurons can cause neuronal apoptosis [60]. The validation of formononetin against two cell damaged models also supported its potential usage in AD therapy. Therefore, our results suggest that the effect of formononetin on AD deserves further study, and it was unexpectedly found that formononetin can improve the nerve damage caused by sodium nitroprusside and potassium chloride in SH-SY5Y cells.

In summary, multiple compounds in HQSJZD are linked to the potential treatment of AD. Among these compounds in HQSJZD, formononetin and hederagenin may be potential lead compounds in the treatment of AD. For the first time, we used virtual screening and network pharmacology to clarify the material basis of HQSJZD in the treatment of AD. This study is expected to broaden the choice of treatment methods for AD and further demonstrate the feasibility of applying network pharmacology to the analysis of TCM prescriptions.

## 5. Conclusions

Our study identified the multi-ingredient and multitarget mechanisms of HQSJZD in the treatment of AD through the network pharmacology method, molecular docking technology, and in vitro experiments and provided a theoretical basis for the development of active compounds of HQSJZD as an innovative alternative therapy for AD.

## Figures and Tables

**Table 1:** Active ingredients of HQ in HQSJZD.

Mol ID	Molecule name	OB (%)	DL
MOL000211	Mairin	55.38	0.78
MOL000239	Jaranol	50.83	0.29
MOL000296	Hederagenin	36.91	0.75
MOL000033	(3S,8S,9S,10R,13R,14S,17R)-10,13-Dimethyl-17-[(2R,5S)-5-propan-2-yloctan-2-yl]-2,3,4,7,8,9,11,12,14,15,16,17-dodecahydro-1H-cyclopenta[a]phenanthren-3-ol	36.23	0.78
MOL000354	Isorhamnetin	49.6	0.31
MOL000371	3,9-Di-O-methylnissolin	53.74	0.48
MOL000378	7-O-Methylisomucronulatol	74.69	0.3
MOL000379	9,10-Dimethoxypterocarpan-3-O-*β*-D-glucoside	36.74	0.92
MOL000380	(6aR,11aR)-9,10-Dimethoxy-6a,11a-dihydro-6H-benzofurano [3,2-c]chromen-3-ol	64.26	0.42
MOL000387	Bifendate	31.1	0.67
MOL000392	Formononetin	69.67	0.21
MOL000417	Calycosin	47.75	0.24
MOL000422	Kaempferol	41.88	0.24
MOL000433	Folic acid	68.96	0.71
MOL000439	Isomucronulatol-7,2′-di-O-glucosiole	49.28	0.62
MOL000442	1,7-Dihydroxy-3,9-dimethoxy pterocarpene	39.05	0.48
MOL000098	Quercetin	46.43	0.28

**Table 2:** Corresponding target of jaranol in HQ.

Mol ID	Molecule name	Target name	Symbol
MOL000239	Jaranol	Nitric oxide synthase, inducible	NOS2
MOL000239	Jaranol	Prostaglandin G/H synthase 1	PTGS1
MOL000239	Jaranol	Androgen receptor	AR
MOL000239	Jaranol	Sodium channel protein type 5 subunit alpha	SCN5A
MOL000239	Jaranol	Prostaglandin G/H synthase 2	PTGS2
MOL000239	Jaranol	Estrogen receptor beta	ESR2
MOL000239	Jaranol	Dipeptidyl peptidase IV	DPP4
MOL000239	Jaranol	Heat shock protein HSP 90-beta	HSP90AB1
MOL000239	Jaranol	Cell division protein kinase 2	CDK2
MOL000239	Jaranol	Serine/threonine-protein kinase Chk1	CHEK1
MOL000239	Jaranol	Trypsin-1	PRSS1
MOL000239	Jaranol	Nuclear receptor coactivator 2	NCOA2
MOL000239	Jaranol	Calmodulin-1	CALM1

**Table 3:** Topological analysis of compound-target network of HQSJZD.

Target	Name	Degree	Radiality	Topological coefficient
A6	Quercetin	304	0.8490814	0.0615844
B1	Kaempferol	188	0.7773403	0.1347009
PTGS2	Prostaglandin g/h synthase 2	109	0.8665792	0.080155
ESR1	Estrogen receptor beta	83	0.7187227	0.1727174
A4	Formononetin	80	0.7493438	0.2999165
AR	Androgen receptor	77	0.8035871	0.1075397
PPARG	Peroxisome proliferator-activated receptor gamma	77	0.8132109	0.1053856
NOS2	Nitric oxide synthase, inducible	74	0.7230971	0.1714154
A3	Isorhamnetin	74	0.7467192	0.3137168
CALM1	Calmodulin-1	74	0.7292214	0.1595632
PTGS1	Prostaglandin g/h synthase 1	62	0.8219598	0.1031328
GSK3B	Glycogen synthase kinase-3 beta	61	0.6749781	0.2467301
ESR2	Estrogen receptor beta	59	0.6741032	0.2473953
MAPK14	Mitogen-activated protein kinase 14	51	0.6662292	0.2610153
D1	Hederagenin	48	0.7353456	0.1575652
A5	Calycosin	46	0.7362205	0.4488294
ADRB2	Beta-2 adrenergic receptor	44	0.7825897	0.1207372
ACHE	Acetylcholinesterase	32	0.7598425	0.1397227

**Table 4:** Lowest binding energy of 6 components of HQSJZD docked with AChE.

Mol ID	Compound name	CAS number	MW	Inhibition (%) against AChE	AChE docking score (Kcal/mol)
ZINC597013	Donepezil	120014-06-4	379.5	90.18	−11.62
MOL000098	Quercetin	117-39-5	302.25	−4.63	−8.38
MOL000422	Kaempferol	520-18-3	286.25	−14.88	−7.98
MOL000392	Formononetin	485-72-3	268.28	60.15	−8.71
MOL000439	Isorhamnetin	480-19-3	316.28	6.35	−8.1
MOL000296	Hederagenin	465-99-6	472.70	45.72	−12.47
MOL000417	Calycosin	20575-57-9	284.26	9.53	−8.68

## Data Availability

The data used to support the findings of this study are available from the corresponding author upon request.
